# Concurrent Superior Mesenteric Artery and Nutcracker Syndromes: A Case Report

**DOI:** 10.7759/cureus.88906

**Published:** 2025-07-28

**Authors:** Ayoub Khaled, Ismail Chaouche, Nizar El Bouardi, Badreddine Alami, Moulay Youssef Alaoui Lamrani, Meryem Boubbou, Mustapha Maaroufi

**Affiliations:** 1 Department of Adult Radiology, Centre Hospitalier Universitaire (CHU) Hassan II, Sidi Mohamed Ben Abdellah University, Fez, MAR; 2 Department of Mother and Child Radiology, Centre Hospitalier Universitaire (CHU) Hassan II, Sidi Mohamed Ben Abdellah University, Fez, MAR

**Keywords:** aortomesenteric angle, gastrointestinal obstruction, left renal vein compression, nutcracker syndrome, superior mesenteric artery syndrome

## Abstract

Superior mesenteric artery syndrome (SMAS) and nutcracker syndrome (NCS) are rare vascular compression disorders caused by entrapment of the third portion of the duodenum and the left renal vein, respectively, between the abdominal aorta and the superior mesenteric artery (SMA). While both conditions share a common pathophysiological mechanism, their simultaneous occurrence remains exceptionally rare. We report the case of a 16-year-old male patient who presented with persistent epigastric pain, nausea, and bilious vomiting. Clinical history revealed recent unquantified weight loss, with a body mass index (BMI) of 17.5 kg/m². Contrast-enhanced computed tomography (CECT) demonstrated a markedly reduced aortomesenteric angle (14.3°) and distance (3.9 mm), consistent with SMAS, along with compression of the left renal vein, upstream dilation, and collateral venous pathways, supporting the diagnosis of concomitant NCS. Due to the severity of duodenal obstruction and failure of conservative management, the patient underwent a transmesocolic anisoperistaltic gastrojejunostomy. Postoperative recovery was uneventful, with complete resolution of gastrointestinal symptoms and satisfactory nutritional improvement at the three-month follow-up. As NCS was asymptomatic, it was managed conservatively with scheduled clinical and radiological surveillance. This case highlights the importance of comprehensive cross-sectional imaging in identifying dual vascular compression syndromes and emphasizes the need for individualized treatment strategies based on symptomatology and anatomical findings.

## Introduction

Superior mesenteric artery syndrome (SMAS) is a rare condition caused by extrinsic compression of the third portion of the duodenum between the abdominal aorta and the superior mesenteric artery (SMA), typically associated with a narrowed aortomesenteric angle of less than 22° [1]. Nutcracker syndrome (NCS), on the other hand, results from compression of the left renal vein (LRV) between the same vascular structures, leading to renal venous hypertension that may clinically manifest as hematuria, orthostatic proteinuria, or left flank pain [2]. Both syndromes share a common anatomical and pathophysiological origin: the loss of retroperitoneal fat between the SMA and the aorta, which predisposes to vascular and duodenal compression [1,2]. Multidetector computed tomography (CT) and other cross-sectional imaging modalities play a pivotal role in diagnosing these conditions and assessing their anatomical and hemodynamic consequences [1,2]. Although SMAS and NCS may theoretically coexist due to their shared etiology, their simultaneous occurrence remains rare and has only been documented in a few isolated cases. One such case was reported by Lao et al., involving a 16-year-old patient with significant weight loss and newly diagnosed Crohn’s disease, in whom both syndromes were identified concurrently [3]. Recognizing this association is essential for accurate diagnosis and appropriate management, as dual vascular compression may exacerbate clinical symptoms and require a multidisciplinary approach.

## Case presentation

A 16-year-old male patient, with no known personal history of chronic illness, prior surgeries, or medication use, presented to the emergency department with a three-day history of persistent epigastric abdominal pain, nausea, and repeated episodes of bilious vomiting. The symptoms had progressively worsened and were not relieved by oral intake or antiemetics. Over the preceding two months, he had experienced an unquantified but noticeable weight loss, estimated by the family at approximately 4 kg, leading to early satiety despite a preserved appetite. His bowel habits were altered, with cessation of bowel movements for two days, although passage of flatus was maintained.

Family history was non-contributory, with no known hereditary gastrointestinal or vascular disorders. On physical examination, the patient appeared asthenic, with a body mass index (BMI) of 17.5 kg/m². He was alert, afebrile (36.8°C), and normotensive (110/70 mmHg) and had a regular heart rate (78 bpm). Respiratory and cardiovascular exams were unremarkable. Abdominal examination revealed a soft, non-distended abdomen without tenderness, palpable masses, guarding, or rebound tenderness. Bowel sounds were hypoactive.

Laboratory investigations revealed normal white blood cell count (7.8 × 10⁹/L), hemoglobin (13.4 g/dL), and C-reactive protein (<2 mg/L). Renal function and electrolyte levels were within normal limits, including serum creatinine (0.9 mg/dL), urea (22 mg/dL), sodium (138 mmol/L), and potassium (4.1 mmol/L). Liver enzymes, aspartate aminotransferase (AST) (22 U/L), alanine aminotransferase (ALT) (25 U/L), and alkaline phosphatase (ALP) (80 U/L), as well as total bilirubin (0.7 mg/dL) and albumin (4.2 g/dL) were also normal. Urinalysis was negative for both hematuria and proteinuria (Table 1).

**Table 1:** Summary of laboratory findings in the reported patient, with corresponding normal reference ranges.

Parameter	Result	Normal range
White blood cell count	7.8 × 10⁹/L	4.0-10.0 × 10⁹/L
Hemoglobin	13.4 g/dL	13-17 g/dL
C-reactive protein (CRP)	<2 mg/L	<5 mg/L
Serum creatinine	0.9 mg/dL	0.6-1.2 mg/dL
Urea	22 mg/dL	10-50 mg/dL
Sodium	138 mmol/L	135-145 mmol/L
Potassium	4.1 mmol/L	3.5-5.0 mmol/L
Aspartate aminotransferase (AST)	22 U/L	10-40 U/L
Alanine aminotransferase (ALT)	25 U/L	10-45 U/L
Alkaline phosphatase (ALP)	80 U/L	40-130 U/L
Total bilirubin	0.7 mg/dL	0.3-1.2 mg/dL
Albumin	4.2 g/dL	3.5-5.0 g/dL
Hematuria (urinalysis)	Negative	Negative
Proteinuria (urinalysis)	Negative	Negative

**Figure 1:**
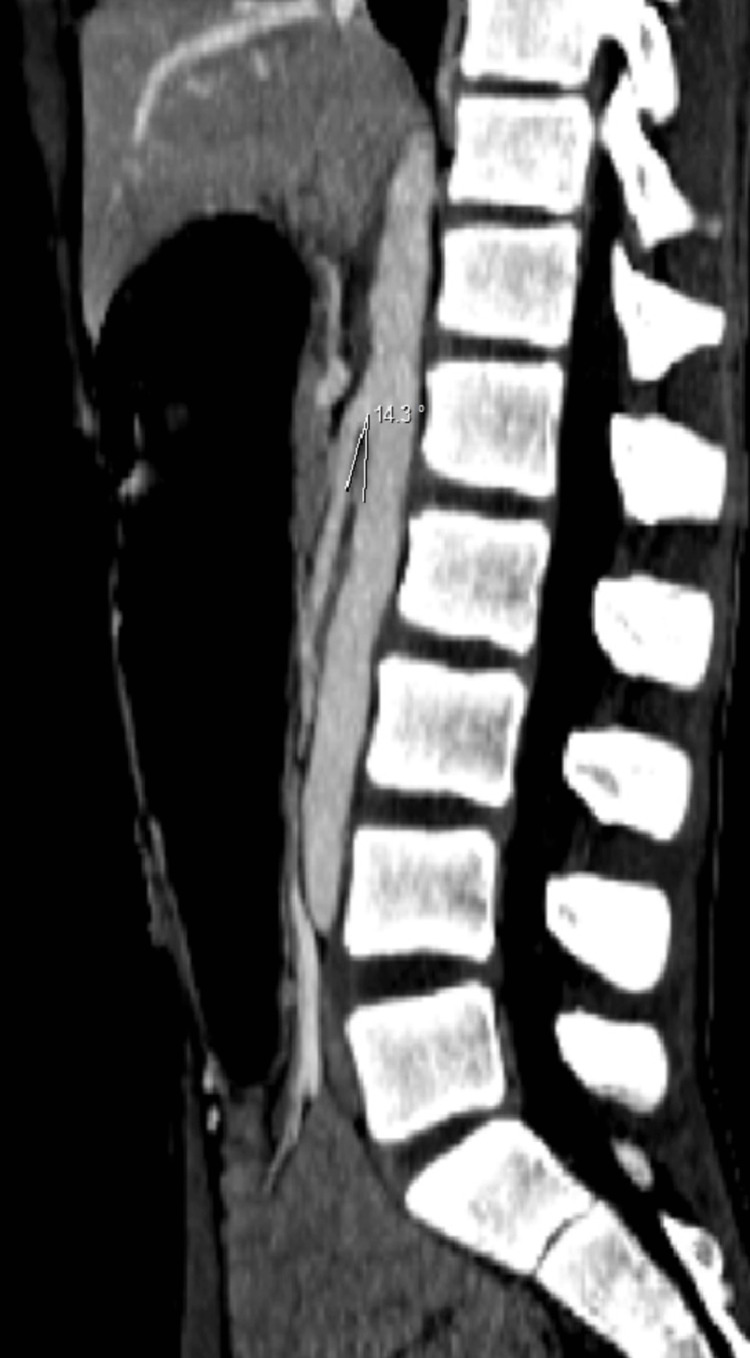
Contrast-enhanced computed tomography (CT); sagittal reconstruction of the abdomen demonstrates a reduced aortomesenteric angle measuring 14.3°.

**Figure 2:**
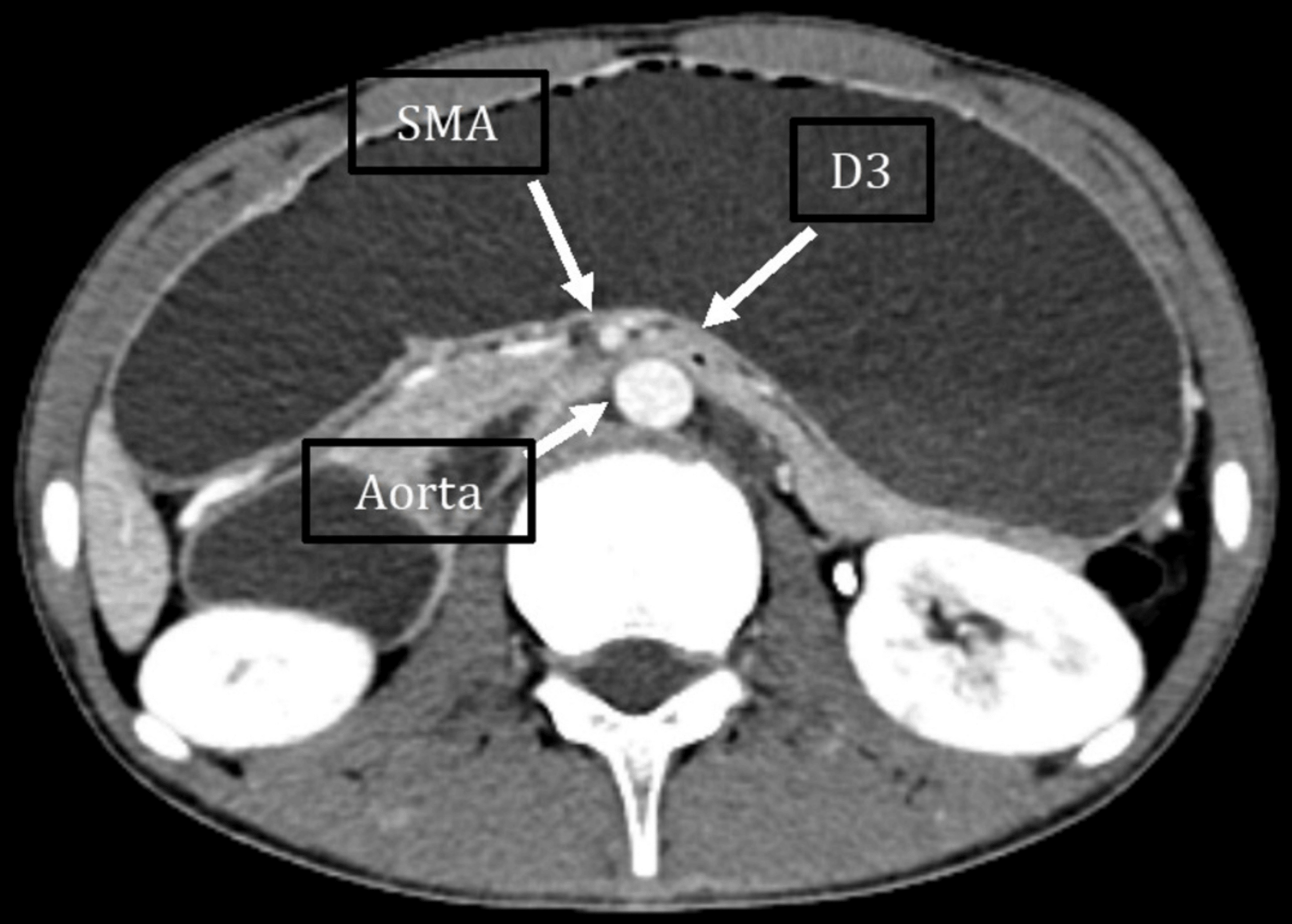
Contrast-enhanced computed tomography (CT); axial reconstruction of the abdomen shows extrinsic compression of the third portion of the duodenum between the superior mesenteric artery (SMA) and the aorta, with the aortomesenteric distance reduced to 3.9 mm.

A contrast-enhanced CT (CECT) demonstrated marked distension of the esophagus, stomach, and proximal duodenum, with a sharp transition at the third portion of the duodenum due to extrinsic compression between the SMA and the abdominal aorta. The aortomesenteric angle was reduced to 14° (Figure 1), and the aortomesenteric distance measured 3.9 mm (Figure 2), consistent with SMAS. The distal small-bowel and colonic loops appeared collapsed.

**Figure 3:**
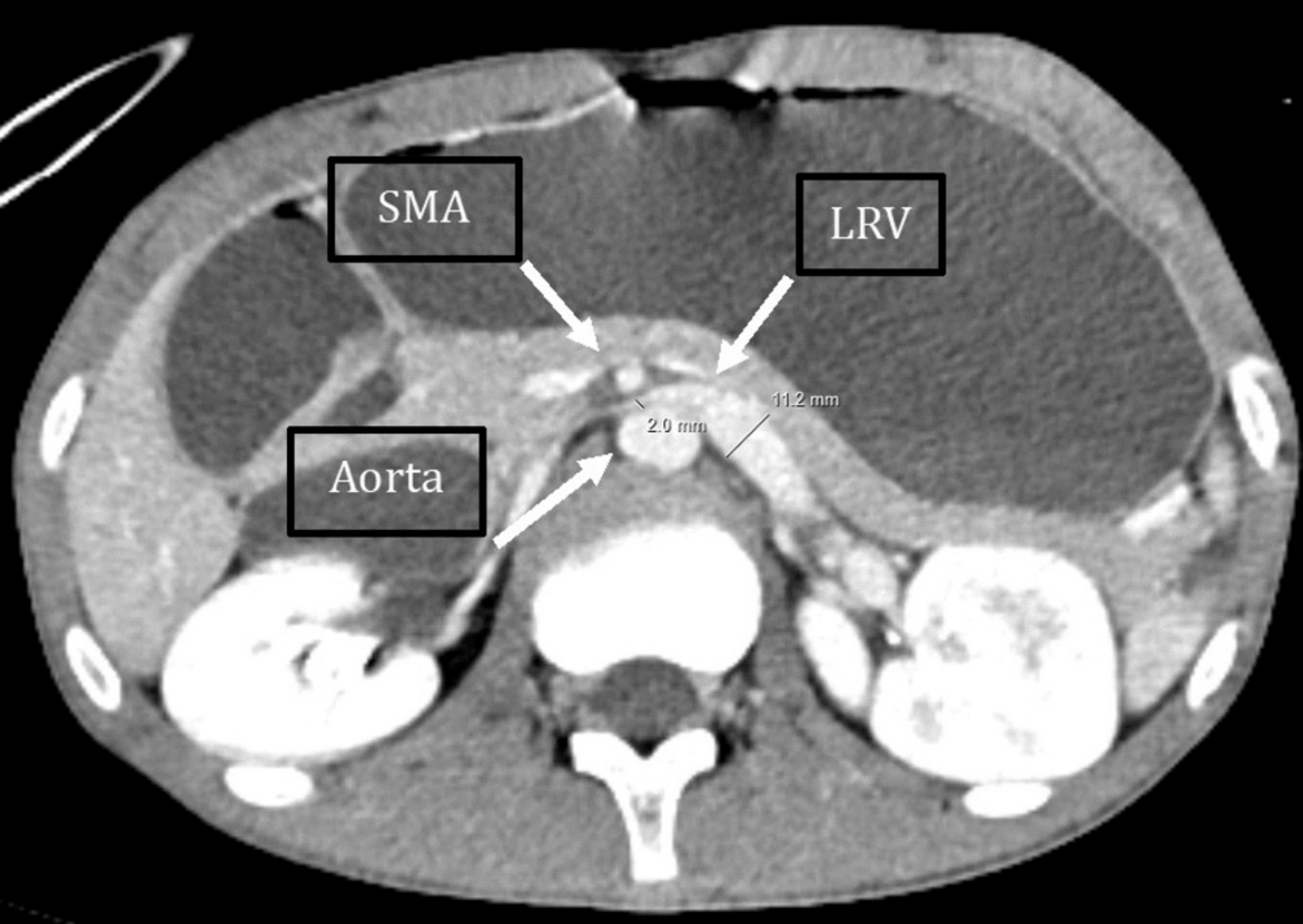
Contrast-enhanced computed tomography (CT); axial reconstruction in the portal venous phase shows narrowing of the left renal vein (LRV) at the level of the aortomesenteric clamp, with proximal venous dilation. SMA: superior mesenteric artery

**Figure 4:**
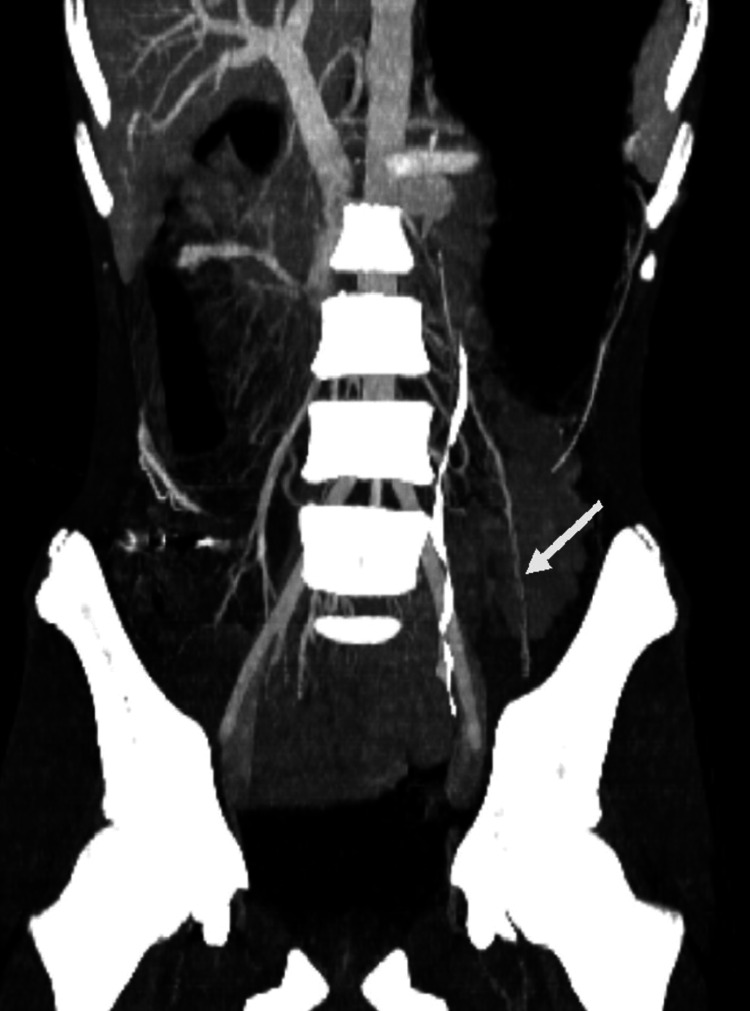
Contrast-enhanced computed tomography (CT); coronal reconstruction in the portal venous phase shows dilation of the left gonadal vein (white arrow).

**Figure 5:**
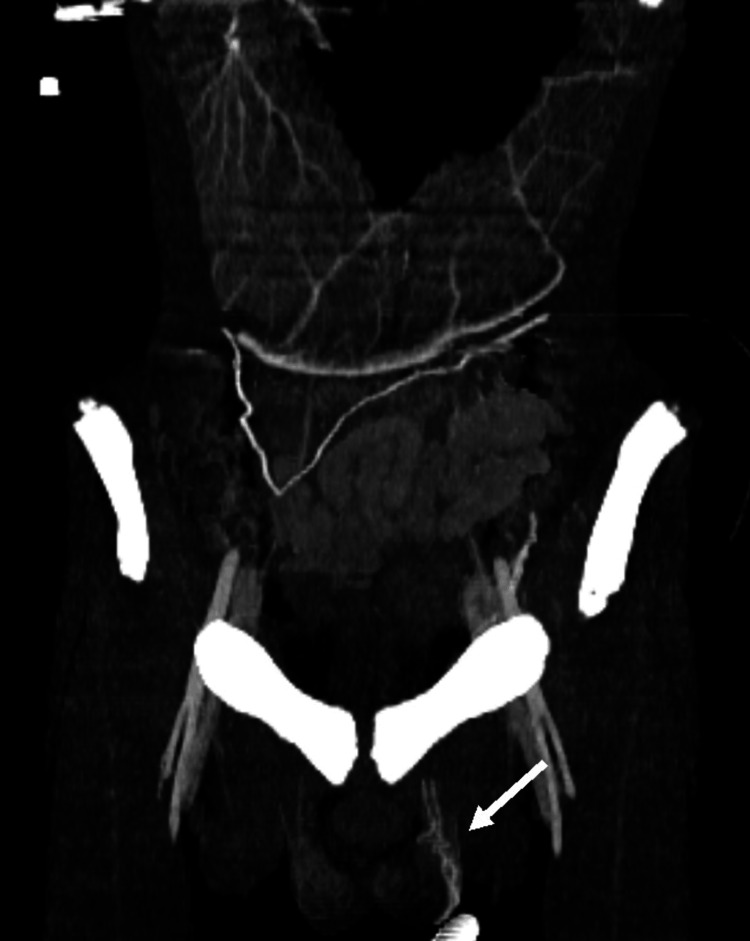
Contrast-enhanced computed tomography (CT); coronal reconstruction in the portal venous phase shows dilation of the left testicular pampiniform plexus (white arrow).

Additionally, the same CT scan demonstrated a narrowed LRV at the aortomesenteric clamp site, with a minimal diameter of 2 mm and significant upstream dilation to 11.2 mm, forming the typical “beak sign” (Figure 3). The hilar-to-aortomesenteric diameter ratio of the LRV was 5.6. Collateral findings included dilation of the left gonadal vein (Figure 4) and the ipsilateral pampiniform plexus (Figure 5), indicating venous congestion and supporting the diagnosis of coexisting NCS (Table 2). 

**Table 2:** Summary of multidetector CT findings in the present case. CT: computed tomography; LRV: left renal vein

Imaging finding	Measurement/description
Aortomesenteric angle	14°
Aortomesenteric distance	3.9 mm
Left renal vein (compressed segment)	2 mm
Left renal vein (dilated segment)	11.2 mm
Hilar-to-aortomesenteric LRV ratio	5.6
Collateral veins	Dilated left gonadal vein and pampiniform plexus

Given the severity of the duodenal obstruction and the failure of conservative treatment, the patient underwent a manual transmesocolic anisoperistaltic gastrojejunostomy. The postoperative course was uneventful, allowing for the gradual reintroduction of oral intake by postoperative day four. He was discharged in stable condition on day seven, with complete resolution of gastrointestinal symptoms. At the three-month follow-up, the patient remained asymptomatic and showed clinical improvement with stabilization of his weight, suggesting satisfactory functional recovery. As the NCS was asymptomatic, it was managed conservatively with scheduled clinical and imaging follow-up.

## Discussion

SMAS, also referred to as Wilkie’s syndrome, is an uncommon but significant clinical entity that results from external compression of the third portion of the duodenum by the SMA as it emerges from the abdominal aorta. This rare anatomical and vascular configuration was first described by Rokitansky in 1861 and later comprehensively detailed by Wilkie in 1921, who contributed to its broader clinical recognition [4]. The syndrome arises due to a narrowing of the aortomesenteric angle and a reduction in the aortomesenteric distance, which together lead to a mechanical obstruction of the duodenum [5].

SMAS predominantly affects adolescents and young adults, with a higher incidence in slender individuals and females. Although its estimated prevalence ranges from 0.013% to 0.3%, the true frequency may be underreported due to misdiagnosis or lack of awareness [4]. Multiple factors contribute to its pathogenesis, the most notable being rapid linear growth during adolescence and significant weight loss, which can result in loss of the retroperitoneal fat cushion that normally prevents vascular compression. Other reported contributing factors include anatomical variants such as a congenitally short ligament of Treitz, spinal deformities, and prior surgical interventions such as scoliosis correction [6,7].

The clinical presentation of SMAS varies widely, ranging from chronic postprandial epigastric pain and early satiety to acute duodenal obstruction with bilious vomiting and nutritional compromise. Diagnosis can be challenging and often delayed due to its non-specific symptomatology. Illustrating this diagnostic delay, Ruhidayati et al. reported that among 35 patients with SMAS who completed a questionnaire, the median interval between symptom onset and initial diagnosis was 22 months (range 3-216 months), and only five patients (14.3%) were diagnosed early [8]. CECT with sagittal reconstruction is the preferred imaging modality, allowing precise measurement of the aortomesenteric angle and distance. A diagnostic threshold of <22° for the angle and <8 mm for the distance is considered highly suggestive of SMAS [9]. Other modalities, such as Doppler ultrasound and magnetic resonance angiography (MRA), can be used adjunctively but are less widely adopted in routine practice.

NCS, on the other hand, refers to the compression of the LRV as it traverses the narrowed space between the aorta and the SMA, leading to renal venous outflow obstruction and venous hypertension. This condition may remain clinically silent or present with symptoms such as gross or microscopic hematuria, orthostatic proteinuria, flank or abdominal pain, pelvic congestion, or left-sided varicocele in men. In rare instances, patients may develop anemia or chronic fatigue due to recurrent bleeding. In a patient-reported cohort described by Charondo et al. (n = 22), 43% reported the onset of symptoms during adolescence, and 62% received their diagnosis in young adulthood. Before achieving an accurate diagnosis, over half were initially evaluated for kidney stones (57%) or ovarian cysts (48%), and most consulted at least 10 to 15 healthcare providers [10]. On imaging, diagnostic features include a narrowed LRV at the aortomesenteric junction (often forming the so-called "beak sign"), proximal venous dilation, and the presence of venous collateral circulation. A hilar-to-aortomesenteric diameter ratio greater than 4.9 and an aortomesenteric angle < 35° further support the diagnosis [11,12].

The coexistence of SMAS and NCS, although anatomically conceivable given their shared pathophysiologic origin-a narrowed aortomesenteric angle-is considered exceedingly rare. The loss of mesenteric fat in asthenic patients predisposes both the duodenum and the LRV to external compression. However, only a limited number of such dual presentations have been reported in the literature. These cases highlight the necessity for radiologists and clinicians to maintain a high index of suspicion and to thoroughly evaluate both gastrointestinal and vascular structures when interpreting imaging studies of patients with compatible symptoms.

Management of SMAS typically begins with conservative treatment, especially in the absence of complete obstruction. Initial measures include nutritional rehabilitation aimed at weight regain, careful caloric monitoring to prevent refeeding syndrome, and enteral feeding via nasojejunal or jejunostomy tubes if oral intake is insufficient. Positional changes such as the prone or left lateral decubitus positions, and maneuvers like the knee-chest or Hayes maneuver (infraumbilical posterior and cephalad compression), have been suggested to enhance enteral feed tolerance. In cases where these approaches fail, a trial of parenteral nutrition may be warranted. Surgical intervention is reserved for patients with persistent or severe obstruction unresponsive to conservative measures. Among surgical options, duodenojejunostomy is widely regarded as the treatment of choice due to its minimal enteric bypass and high success rates reported in case series. Gastrojejunostomy, which is associated with greater nutritional loss, is typically considered a second-line option. Strong’s procedure, involving division of the ligament of Treitz and derotation of the duodenum, has demonstrated a relatively high failure rate, likely due to persistent duodenal entrapment between the pancreaticoduodenal arteries. Given the rarity of the condition and the absence of large clinical trials, there is currently no level 1 evidence guiding surgical management [13].

Management of NCS is symptom-driven: asymptomatic or mildly symptomatic patients are observed with clinical and imaging follow‑up, whereas debilitating symptoms warrant LRV decompression, typically by vein transposition, endovascular stenting, or renal autotransplantation; laparoscopic extravascular stent placement (e.g., titanium/polyetheretherketone (PEEK)) is a minimally invasive, promising option but still lacks robust long-term comparative evidence (Table 3) [14,15].

**Table 3:** Comparison of nutcracker syndrome and superior mesenteric artery syndrome: mechanism, imaging features, and management strategies CT: computed tomography; SMA: superior mesenteric artery

Feature	Superior mesenteric artery syndrome (SMAS)	Nutcracker syndrome (NCS)
Mechanism	Duodenum (3rd portion) compressed between SMA and aorta	Left renal vein compressed between SMA and aorta
Typical clinical presentation	Postprandial epigastric pain, early satiety, vomiting, weight loss	Hematuria, flank pain, pelvic congestion, varicocele, fatigue
Imaging signs	Aortomesenteric angle < 22°, distance < 8 mm (CT sagittal views)	“Beak sign,” proximal venous dilation, venous collateral circulation, hilar-to-aortomesenteric diameter ratio > 4.9
First-line management	Conservative: nutritional rehabilitation (high-calorie, enteral feeding), postural measures	Conservative if asymptomatic or mildly symptomatic: observation and follow-up imaging
Surgical management	Duodenojejunostomy is the preferred surgical option in refractory cases. Gastrojejunostomy is second-line. Strong’s procedure has a high failure rate and is rarely used	Vein transposition, endovascular stenting, or renal autotransplantation; laparoscopic extravascular stenting is a promising alternative

In the case we report, the patient presented with a clinically significant SMAS requiring surgical management, which led to rapid and sustained symptom resolution. The associated NCS was incidentally discovered and asymptomatic at the time of diagnosis. Consequently, it was managed conservatively with scheduled clinical follow-up and surveillance imaging. This dual presentation reinforces the importance of a comprehensive and methodical radiologic approach in identifying multiple concurrent vascular compression syndromes, especially in young, underweight patients with upper gastrointestinal obstructive symptoms.

Learning point

In any suspected SMAS, always measure the aortomesenteric angle and distance and scrutinize the LRV for entrapment to exclude a combined compression syndrome; multidisciplinary input from radiology, gastroenterology, and vascular specialties is often essential for accurate diagnosis and optimal management.

## Conclusions

The simultaneous occurrence of SMAS and NCS is exceptionally uncommon, yet both entities share the same pathophysiological substrate-a markedly reduced aortomesenteric angle that compresses the third portion of the duodenum and entraps the LRV. Contrast-enhanced cross-sectional imaging, chiefly multidetector CT, remains the reference modality, as it provides a comprehensive assessment of gastrointestinal obstruction and associated vascular compressions in a single examination.

This case highlights the imperative of meticulous multiplanar imaging analysis when confronted with proximal duodenal obstruction, particularly in cachectic patients or following rapid weight loss. Equally important is the systematic evaluation for concurrent vascular anomalies-most notably NCS when hematuria is unexplained or a left-sided varicocele is present-to secure an accurate diagnosis and tailor management.
